# Biological Functions Driven by mRNAs Carried by Extracellular Vesicles in Cancer

**DOI:** 10.3389/fcell.2021.620498

**Published:** 2021-08-30

**Authors:** Marta Prieto-Vila, Yusuke Yoshioka, Takahiro Ochiya

**Affiliations:** Department of Molecular and Cellular Medicine, Institute of Medical Science, Tokyo Medical University, Tokyo, Japan

**Keywords:** cancer, mRNA, extracellular vesicles, biological function, exosome, EV

## Abstract

Extracellular vesicles (EVs), including exosomes and microvesicles, are extracellular nanovesicles released by most cells. EVs play essential roles in intercellular communication *via* the transport of a large variety of lipids, proteins, and nucleic acids to recipient cells. Nucleic acids are the most commonly found molecules inside EVs, and due to their small size, microRNAs and other small RNAs are the most abundant nucleic acids. However, longer molecules, such as messenger RNAs (mRNAs), have also been found. mRNAs encapsulated within EVs have been shown to be transferred to recipient cells and translated into proteins, altering the behavior of the cells. Secretion of EVs is maintained not only through multiple normal physiological conditions but also during aberrant pathological conditions, including cancer. Recently, the mRNAs carried by EVs in cancer have attracted great interest due to their broad roles in tumor progression and microenvironmental remodeling. This review focuses on the biological functions driven by mRNAs carried in EVs in cancer, which include supporting tumor progression by activating cancer cell growth, migration, and invasion; inducing microenvironmental remodeling *via* hypoxia, angiogenesis, and immunosuppression; and promoting modulation of the microenvironment at distant sites for the generation of a premetastatic niche, collectively inducing metastasis. Furthermore, we describe the potential use of mRNAs carried by EVs as a noninvasive diagnostic tool and novel therapeutic approach.

## Introduction

Intercellular communication is essential for the appropriate performance of multicellular organisms. Recently, extracellular vesicles (EVs) have emerged as a novel mechanism of horizontal gene transfer. EVs are lipid bilayer membrane-enclosed vesicles released by most, if not all, cell types. They carry a wide range of fragile RNA molecules, DNA molecules and proteins within their core (Valadi et al., 2007; Skog et al., 2008; Balaj et al., 2011; Li et al., 2013; Raposo and Stoorvogel, 2013). These molecules are protected by EV membranes from nucleases, proteases, fluctuations in pH and osmolality, and other environmental factors that would otherwise rapidly degrade them (El-Hefnawy et al., 2004; Skog et al., 2008; Cheng et al., 2014; Kim et al., 2017). Thanks to this protection, the cargo is successfully transported, enters into recipient cells, and affects cell behavior, having a significant impact on natural physiological processes. Thus, EVs are considered important mediators of intercellular communication, both in physiological and pathological states, including cancer (Kosaka et al., 2013b; Berardocco et al., 2017; Kim et al., 2017).

### The Short History of Exosomes

Exosomes were first observed in the early 1980s by the Stahl and Johnstone groups—who published within a week of each other—in a study regarding the maturation of reticulocytes into erythrocytes by the removal of transferrin receptors (Pan and Johnstone, 1983; Harding et al., 1984). In that study, it was observed that transferrin receptors were internalized within multivesicular bodies (MVB) and that later, instead of being degraded, they were externalized. At that time, exosomes were described as a mechanism to discard unwanted cellular debris (Johnstone et al., 1987; Harding et al., 2013). One decade later, Raposo et al. (1996) found that exosomes derived from B lymphocytes activated T lymphocytes. Soon thereafter, it was shown that dendritic cells also produced EVs (Raposo et al., 1996; Théry et al., 2001), suggesting a role for EVs in antigen presentation. Later research identified messenger RNA (mRNA) and microRNA (miRNA) as exosome cargo and recognized their transfer into recipient cells, suggesting the biological activity of EVs (Ratajczak et al., 2006; Valadi et al., 2007; Wysocynski and Ratajczak, 2009; Raposo and Stoorvogel, 2013) and revealing their role as essential cell-to-cell communication vehicles.

### Types of Extracellular Vesicles

The International Society for Extracellular Vesicles (ISEV) was founded in 2012. This entity provided the criteria to classify EVs into three groups according to their biogenesis: exosomes, microvesicles (MVs), and apoptotic bodies (AB) (Gould and Raposo, 2013; Yáñez-Mó et al., 2015; Théry et al., 2018).

Among the three subtypes, exosomes are the most thoroughly studied. Exosomes are the smallest of the subtypes, with a diameter of between 30 and 150 nm, and are primarily formed *via* an endocytic pathway. Endocytic invagination forms early endosomes (Bissig and Gruenberg, 2013), and during their maturation into MVB, inward budding results in the generation of intraluminal vesicles (ILVs). In this process, cytosolic RNAs are taken up into ILVs. Because ILVs become exosomes, this inward budding process is designated “RNA loading into exosomes” (Janas et al., 2015). Then, most MVBs are directed to lysosomes for degradation. However, a subset of MVBs moves along microtubules toward the cell membrane by several Rab-GTPase molecules (Fevrier et al., 2004). When it reaches the cell membrane, the limiting membrane of the MVB fuses with the plasma membrane, and ILVs are released into the extracellular space (Pant et al., 2012; Yáñez-Mó et al., 2015). After secretion, ILVs are termed exosomes. As a consequence of their origin, exosomes contain a set of specific proteins and lipids. For instance, exosomes contain endosome-associated proteins involved in MVB biogenesis and RNA sorting, such as the Endosomal Sorting Complex Required for Transport (ESCRT) complex, Alix and Tsg101 (Nabhan et al., 2012). Other exosome markers are the tetraspanins CD9, CD63, and CD81 (Kowal et al., 2014). Although exosomes are derived from the cell membrane, they are formed during the spontaneous inward budding of raft-like regions, and therefore, their lipid content is slightly different from that of the cell membrane. Exosomes are enriched in cholesterol, sphingomyelin, glycosphingolipids, and phosphatidylcholine with saturated fatty acids (Laulagnier et al., 2004; Raposo and Stoorvogel, 2013; Janas et al., 2015).

Microvesicles have a diameter between 100 and 1000 nm (Bobrie et al., 2011). In contrast to exosomes, MVs are produced directly from the cell surface by outward budding and fission of the plasma membrane. Hence, their surface markers and lipids are primarily dependent on the composition of the cell membrane (Ratajczak et al., 2006; Lee et al., 2012). MVs also contain transmembrane proteins commonly found in the cell membrane, such as integrins and selectins (Bobrie et al., 2011; Raposo and Stoorvogel, 2013).

Apoptotic bodies are the largest EVs, 1–5 μm in diameter on average. These vesicles are products of cell disassembly during apoptosis and are released *via* blebbing of the plasma membrane (Ivan et al., 2014). Their cargo is quite distinct from that of exosomes and MVs, since in addition to proteins, lipids, DNA, and ribosomal RNA (rRNA), they harbor cytoplasm, organelles and histones with or without a nuclear fragment (Ivan et al., 2014; Van Niel et al., 2018).

**FIGURE 1:**
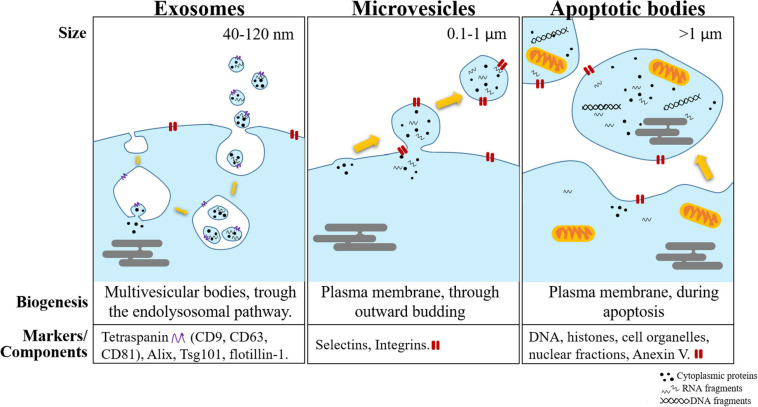
Schematic representation of the different subtypes of extracellular vesicles (exosomes, microvesicles, and apoptotic bodies). These vesicles are separated mainly by their size, biogenesis, and the most common markers used to identify them.

A summary of the comparison among the three subtypes of EVs is shown in Figure 1.

Differential ultracentrifugation is the most commonly used approach for EV purification (Théry et al., 2006). Ultracentrifugation can remove soluble components from EV preparations; however, similarly sized particles (membrane vesicles and protein aggregates) copurify (Vergauwen et al., 2017). Therefore, due to the overlap in size between MVs and exosomes, common methods of vesicle isolation do not allow the proper separation of these two populations. The use of markers to separate these two populations is also unreliable since there is still a lack of specific markers to distinguish these populations (Tauro et al., 2012). Furthermore, exosomes and MVs, to a large extent, have a similar molecular composition (Lee et al., 2012; Crescitelli et al., 2013). For these reasons, in this article, we review all articles mentioning both exosomes and MVs. To unify the nomenclature throughout this review, we refer to them collectively as EVs, a term that has been recommended by the ISEV for use as a generic term (Théry et al., 2018; Witwer and Théry, 2019).

### The Importance of Extracellular Vesicles in Cancer

A large body of evidence has shown that all organisms, despite their complexity and diversity, secrete EVs. This secretion appears to be a conserved mechanism across all three domains of living organisms: Archaea, Bacteria, and Eukarya (Gill et al., 2019). EVs have been shown to mediate cell–cell communication, playing a key role in the regulation of various physiological processes and the pathogenesis of many diseases, being one of them, cancer (Jasmina et al., 2008; Kosaka et al., 2013b, 2016). Cancer cells secrete EVs to both cells in proximity and distal sites, transferring a variety of molecules that modify the microenvironment, which in turn favors cancer cell growth, invasion, and metastasis (Skog et al., 2008; Hood et al., 2011; Yang and Robbins, 2011; Peinado, 2012; He et al., 2015). The amount of shed EVs rises sharply in disease states compared with nondisease states (Knijff-Dutmer et al., 2002; Noerholm et al., 2012). The interest of scientists and physicians in EVs has increased logarithmically in response to the discoveries that EVs can be isolated from body fluids such as serum, plasma, urine, and cerebrospinal fluid and can be used as biomarkers (Lee et al., 2012; Raposo and Stoorvogel, 2013; Tewari, 2015). Here, we review the biological effects of EVs and, more specifically, their mRNA cargo in the context of human cancers. We will further discuss their involvement in tumor progression, invasion, microenvironmental remodeling, and metastasis.

## RNA Content in EVs

Extracellular vesicles contain an abundant cargo of different RNA species that can modulate physiological responses in recipient cells. Since EVs have a small size in comparison to cells, it is easy to understand that most previous studies on EV content focused on miRNAs and other smaller RNA species, such as Y-RNA, rRNA, transfer RNA (tRNA), piwi-interacting RNA (piRNA), and small nucleolar RNA (snoRNA) (Kosaka et al., 2010; Nolte’T Hoen et al., 2012; Lefebvre et al., 2016; Wei et al., 2017). However, larger RNA species, including mRNA, long non-coding RNA (lncRNA), and circular RNA (circRNA), have also been described in EVs (Valadi et al., 2007; Nolte’T Hoen et al., 2012; Dong et al., 2016; Lefebvre et al., 2016). Despite efforts to describe the different RNA species and their abundance in EVs, this topic remains controversial. For instance, while Bellingham et al. (2012) described that close to 90% of the RNA content was tRNA, Wei et al. (2017) only found between 11.6 and 17.4%, depending on the EV subtype (exosome and MV, respectively). Nonetheless, this value was even lower in the study done by Huang et al. (2013), in which they found only 1.24% of tRNA. Similar discrepancies in amount have been described mainly with microRNA and rRNA (Nolte’T Hoen et al., 2012; Huang et al., 2013; Wei et al., 2017). These dissimilarities are mainly due to the differences in the sequencing methodologies, the species of EVs analyzed, and the cell type of origin. However, it seems to be the consensus that smaller RNAs are more abundant than larger species, especially in exosomes compared with MVs (Dong et al., 2016; Lefebvre et al., 2016; Wei et al., 2017). This pattern makes sense since those types of EVs are also smaller than the other types and their biogenesis is different.

Due to the fragile nature of RNA and the large amount of ribonucleases (RNases) in the extracellular milieu, naked RNA fragments are rapidly degraded. However, the ability to detect RNAs in serum suggests that they are found in structures and protected from degradation. Those structures include Argonaute2 complexes and EVs (Arroyo et al., 2011). miRNAs encapsulated in EVs can resist digestion by RNases, with a decrease in the RNA content of less than 7%, showing that most miRNAs are inside EVs (Skog et al., 2008). This protection by the EV membrane is especially important for mRNAs, since compared to miRNAs, they are longer and are therefore more fragile.

### mRNA Loading Into EVs

It has been shown that mRNA and other small RNAs loading is not a passive event but rather a selective process (Guduric-fuchs et al., 2012). Although the mRNAs carried by EVs are the same as those that are expressed in the cell of origin and the cargo of EVs is thus representative of their cell of origin (Jenjaroenpun et al., 2013), it has been found that the relative amount of each mRNA is not completely correlated (Nolte’T Hoen et al., 2012). The similitude with the cellular content is higher in MVs rather than exosomes (Wei et al., 2017). Many RNAs isolated from EVs were found to be enriched in EVs in relation to the RNA profiles of the cells of origin, while others were specifically excluded or not found at all. This indicates that RNA molecules are selectively incorporated into EVs (Skog et al., 2008; Nolte’T Hoen et al., 2012; Kim et al., 2017). For instance, Hong et al. (2009) reported that despite the similarities at the global level between EVs and cellular mRNAs, they identified 241 mRNAs enriched and 1461 diminished in EVs. Moreover, of the enriched mRNAs, 27 were involved in the cell cycle, suggesting that EV shedding might be cell cycle-dependent (Hong et al., 2009). Specifically, packaging during the S phase of cell division has been proposed due to the specific transcript content in EVs (Conley et al., 2017).

The enrichment of certain types of mRNA has also been explained by zip codes. Zip-codes are sequences typically found in the 3′-untranslated regions (3′UTRs) of mRNA transcripts that mediate the binding of a ribonuclear protein complex and eventually mediate mRNA localization within EVs (Martin and Ephrussi, 2009). A 25 nt sequence with a “CTGCC” core sequence was found in all mRNAs highly enriched in EVs derived from glioblastoma (GBM) and melanoma cells. Interestingly, this zip code could be used to increase the levels of mRNAs in EVs, as shown by the increased amount of GFP mRNA, while a mutation in the core sequence inhibited the loading (Bolukbasi et al., 2012). A similar zip code was found in miRNAs and called EXOmotif; consisting of the sequence “GGAG” (Villarroya-Beltri et al., 2013). Furthermore, the sequence “UGCA” was found in miRNAs specifically excluded from EVs and was called the CLmotif (Villarroya-Beltri et al., 2013). These motifs, in both mRNAs and miRNAs, control their loading into EVs, modulating the cargo in these vesicles.

Certain proteins, such as ESCRT, are well-known machinery for the loading of several molecules into EVs (Kowal et al., 2014). More specifically, the so-called Arc proteins assist in mRNA transfer into EVs through ionic interactions with this nucleic acid molecule (Pastuzyn et al., 2018). Other proteins compressed within the autophagy pathway, such as the LC-3/ATG8 complex, were found to regulate the loading of several RNAs into EVs (Leidal et al., 2020). LC-3 protein interacts with RNA-binding proteins (RBP), which at the same time, bind to snoRNA increasing their loading. Interestingly, this complex also required neutral sphingomyelinase 2 (nSMase2), another protein known to regulated EV secretion by the synthesis of ceramides (Trajkovic et al., 2008). However, many important mechanisms for mRNA packaging remain unknown.

### mRNA Fragmentation

Whether the various RNA molecules in EVs are intact or partially degraded is an essential consideration, because intact mRNAs could act as templates for functional proteins in recipient cells. Although EVs contain long RNA molecules, their average length is shorter than that of RNA molecules found within cells. It was found that the cargo of EVs derived from hepatocellular carcinoma cells spanned a range of between 25–50 and 4000 nt (He et al., 2015; Berardocco et al., 2017). These lengths are considerably different from those of cellular mRNAs, which have lengths of between 400 and 12,000 nt (Batagov and Kurochkin, 2013; Enderle et al., 2015). One possible explanation for these observations could be that exosomes are enriched in mRNAs encoding very short proteins. In addition, fragmented mRNA has been found in EVs, along with full-length mRNA (Batagov and Kurochkin, 2013). In contrast to the initial view, both full-length and truncated mRNAs are transferred into recipient cells and can be translated into proteins, but the resulting proteins have different functions (Batagov and Kurochkin, 2013). Fragmented mRNAs are translated mainly into enzyme modulators and proteins participating in extracellular transport, while full-length mRNAs are translated into cell surface receptor, cell communication and system development proteins (Batagov and Kurochkin, 2013). The difference in function suggests a specialization mechanism rather than a random phenomenon. Indeed, mRNA fragments and their loading into EVs was correlated with the proximity of the probes to the 3′UTRs of their transcripts. The 3′UTRs of mRNAs are rich in regulatory sequences and seem to also be involved in the translational ability of mRNA fragments.

## mRNAs Are Functional in Recipient Cells

Extracellular vesicle mRNAs are not only stable mRNA molecules that are accessible for use as biomarkers, they can also be delivered to recipient cells and be translated into functional proteins (Valadi et al., 2007). EVs were first described as a cellular mechanism to discard unwanted cellular debris (Johnstone et al., 1987; Harding et al., 2013), and therefore, they were thought to not have other functions. A few years later, the antigen-presenting function of B lymphocyte-derived EVs to activate T cells was described, followed by the equivalent activation by dendritic cell-derived EVs. Therefore, it was thought that EVs played a role in the immune system (Bobrie et al., 2011). It was not until the reports by Ratajczak et al. (2006) and Valadi et al. (2007) the function of mRNAs contained in EVs and their ability to be translated into proteins was demonstrated.

Ratajczak et al.’s (2006) provided the first evidence regarding the function of mRNAs transferred to recipient cells, that is to be translated into proteins. They found that embryonic stem (ES) cells and their derived EVs contained high levels of *Wnt-3* mRNA. When parental cells were cultured with ES cell-derived EVs, several stemness-related genes were upregulated (Ratajczak et al., 2006). In another study using mouse mast cell-derived EVs and the machinery in rabbit reticulocyte lysates as an *in vitro* translation system, mouse-derived proteins (COX5B, HSPA8, SHMT1, LDH1, ZFP125, GPI1, and RAD23B) were detected by mass spectrometry analysis, confirming the possibility of the translation of EV-derived mRNAs (Valadi et al., 2007). However, Skog et al. (2008) showed the complete process. GBM cells were transduced with a lentiviral vector encoding a secreted luciferase from *Gaussia* (Gluc) (Tannous et al., 2005), and EVs from these cells were collected and confirmed to contain the mRNA but not the protein. When those EVs were added to endothelial cells (ECs), Gluc activity produced by recipient cells was detected, confirming that the mRNA delivered into recipient cells generated a functional protein (Skog et al., 2008). Similar studies have used GFP instead of Gluc, allowing detection of transfected cells by confocal microscopy after a 6-h incubation (Deregibus et al., 2007). Moreover, to further confirm that mRNA derived from EVs and not other elements within the EVs were the main effector of function, mRNA was isolated from EVs and transfected into recipient EC with lipofectamine (Deregibus et al., 2007). Overall, all these *in vitro* assays showed that EV mRNA is functional in recipient cells, leading to behavioral changes in recipient cells *in vitro*.

The following step should then be to assess the translational capacity *in vivo*. Zomer et al. (2015) elegantly demonstrated the transfer of functional cargo in EVs *in vivo* using the Cre-LoxP system from an *a priori*-defined tumor cell population. The Cre-LoxP system induced a color switch (from red to green) in cells that internalized EVs released from specific cells expressing Cre recombinase (Ridder et al., 2014). The switch could be observed by *in vivo* imaging. They demonstrated that highly malignant cancer cells communicated with their microenvironment and other less malignant cancer cells and induced, through transfer of EVs, increases in migratory behavior and metastatic capacity (Zomer et al., 2015). *Via* this system, the authors also demonstrated that although B16 melanoma cells had the ability to take up EVs from healthy cells, this transfer was not frequent. The opposite phenomenon—transfer from cancer cells to healthy cells—was more common.

A summarized information of all the events can be found in Table 1.

**TABLE 1:** Historiological order of essential research to prove functional loading of mRNA into recipient cells *via* EVs.

**Year**	**Finding**	**Experiment**	**References**
2006	Proposal of the effect of mRNA carried by EVs.	ES-derived EVs containing high levels of Wnt3 induced stemness-related gene expression in recipient cells.	Ratajczak et al., 2006
2007	Translation of mRNA is possible.	Mouse mRNA from EVs was translated with the machinery in rabbit reticulocyte lysates.	Valadi et al., 2007
2008	mRNA was transcribed in recipient cells *in vitro*.	EVs with Gluc mRNA was transcribed in recipient EC and generated Gluc signal.	Skog et al., 2008
2015	mRNA was transcribed in recipient cells *in vivo*.	Using the CRE-LoxP system, a switch in color was found in recipient cells in mice.	Zomer et al., 2015

## Functions of EVs in Cancer

Extracellular vesicles play an essential role in cellular communication under both physiological and pathological conditions. However, cancer cells constitutively secrete EVs in greater numbers than their healthy counterparts (Knijff-Dutmer et al., 2002; Noerholm et al., 2012). This pattern can be inferred from biological fluids obtained from patients with cancer. For instance, a study reported that while the blood of healthy patients contained approximately 2 × 10^12^ EVs/mL, the blood of cancer patients contained 4 × 10^12^ EVs/mL (Kalluri, 2016), although further studies are needed to confirm these values since other reports showed the contrary effect (Fang et al., 2016). Nonetheless, it has been shown that under stress, cells increase the secretion of EVs (Harmati et al., 2019; O’Neill et al., 2019). Such stress could be hypoxia (King et al., 2012), laser irradiation inducing autophagy (Bagheri et al., 2018), or oxidative stress (Harmati et al., 2019). The tumoral microenvironment contains most of the cellular stresses mentioned before and some more, including low levels of oxygen, nutrients, low pH, oxidative stress, and others (Chen and Xie, 2018). These factors could explain the general tendency of an increased EV secretion. Importantly, since EVs can mirror their cell of origin to a certain extent, their cargo is oncogenic. Tumor-derived EVs ensure crosstalk between tumor cells and their microenvironment for the benefit of cancer cells. Tumor-derived EVs have been reported to play major roles in the modulation of tumorigenesis, angiogenesis, the immune response, drug resistance, and invasion. Moreover, since EVs are secreted in large amounts into the bloodstream, they can travel to distant sites, where they promote premetastatic niche formation, extravasation, and the survival of metastatic cells at these distant sites (Hood et al., 2011; Peinado, 2012; Hoshino et al., 2015; Tominaga et al., 2015; Yokoi et al., 2017; Costa-silva et al., 2018).

### Tumor Microenvironment

Tumors are not composed only of cancer cells; they are located within an environment containing numerous types of stromal cells, including fibroblasts, EC, mesenchymal cells, and immune cells. This environment is called the tumor microenvironment and plays multiple roles in tumor progression (Whiteside, 2008). The tumor microenvironment also includes the extracellular matrix and factors secreted by these cells, such as growth factors, cytokines, and EVs (Whiteside, 2008; Hu et al., 2018). Cancer cells release EVs into the tumor microenvironment, and these EVs are taken up by other cells, altering their signaling pathways and gene regulation programs. Thus, EVs reshape the local tumor environment into a more favorable niche for tumor growth, invasion, and metastatic spread (Taylor and Gerçel-Taylor, 2005; Skog et al., 2008; Wysocynski and Ratajczak, 2009; Peinado, 2012; Salomon et al., 2013; He et al., 2015; Costa-silva et al., 2018). *Via* these mechanisms, EVs can promote EC growth and thus induce tumor angiogenesis (Deregibus et al., 2007), cause immune tolerance by activating suppressive pathways in immune cells (Valenti et al., 2007), transform fibroblasts into cancer-associated fibroblasts (Webber et al., 2010) and directly modify and disrupt the extracellular matrix (Dolo et al., 1999). Importantly, since EVs are secreted into body fluids, they can also reach distal organs and affect the environment, facilitating metastasis at those sites (Hood et al., 2011; Peinado, 2012; Tominaga et al., 2015; Yokoi et al., 2017; Costa-silva et al., 2018).

**FIGURE 2:**
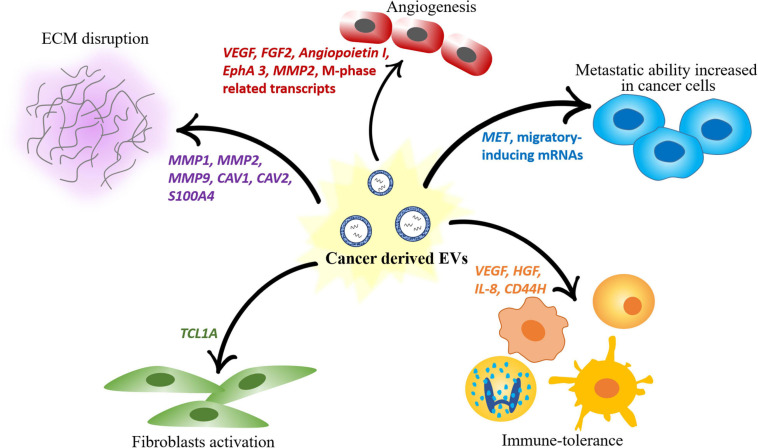
Scheme of the different roles of mRNAs contained in EVs in modulating the components of the tumor microenvironment. Cancer-derived EVs induce angiogenesis, disrupt the ECM, promote fibroblast activation and immune tolerance and increase the metastatic ability of other cancer cells.

Following, several biological functions activated by the different tumor microenvironment components as a response to cancer cell-derived EVs are described. A summary of the same processes is provided in Figure 2.

#### Angiogenesis

Angiogenesis is the formation of new blood vessels from a preexisting vascular network by the proliferation of ECs, which occurs naturally during organismal growth and development and during tissue restoration. Angiogenesis is a vital process for the development of tumors, supplying cancer cells with the nutrients, and oxygen necessary for their growth (Carmeliet, 2005). The newly formed tubes also provide a route for metastatic cells to escape (Nishida et al., 2006; Kikuchi et al., 2019). Therefore, cancer cells, especially those in rapidly growing solid tumors, promote angiogenesis (Carmeliet, 2005; Todorova et al., 2017).

Paradoxically, hypoxia is a common feature in many cancers (Finger and Giaccia, 2010). Angiogenesis and hypoxia frequently co-occur. Under hypoxic conditions, cells secrete multiple proangiogenic molecules that facilitate the proliferation of ECs toward the tumor. Among the large variety of proangiogenic molecules, which commonly include cytokines and growth factors directly secreted into the extracellular space, EVs, and their cargo have also been shown to favor tumor vascularization (Salomon et al., 2013; Kikuchi et al., 2019). However, it is especially during hypoxia that this process is enhanced (Wysocynski and Ratajczak, 2009). There is evidence in several systems for boosted EV release under hypoxic (King et al., 2012) and anoxic conditions (Gupta and Knowlton, 2007). Developmental studies have shown that the release and bioactivity of placental mesenchymal stem cell (pMSC)-derived EVs is oxygen concentration-dependent. Under hypoxic conditions (1 or 3% O_2_), EV release from pMSCs increased by up to sevenfold compared to that from cells incubated under normoxic conditions (Salomon et al., 2013). Similarly, exposure of breast cancer cells to moderate (1%) and severe (0.1%) hypoxia resulted in increases of 30 and 90%, respectively, in the number of particles in the conditioned medium. EV secretion is regulated by the transcriptional factor HIF-1α (King et al., 2012); however, the specific mechanisms by which hypoxia induces EV release remain to be clarified.

As mentioned, angiogenesis is a common process under physiological conditions and occurs during the whole lifespan of organisms. Developmental studies have shown that pMSC-derived EVs promote EC migration and the formation of capillary-like structures *in vitro* (Salomon et al., 2013). In adult organisms, cells with some extent of stemness have also been proven to induce angiogenesis. EVs derived from endothelial progenitor cells (EPCs) induced proliferation and *in vitro* tube formation by horizontal transfer of mRNA associated with the PI3K/AKT signaling pathway (Deregibus et al., 2007). The PI3K/AKT pathway promotes EC proliferation, a requirement for angiogenesis (Karar and Maity, 2011). Indeed, this important pathway was also activated by endothelial colony-forming cell-derived EVs. In this situation, the authors found angiogenesis stimulation both *in vitro* and *in vivo* through the delivery of mRNAs associated with eNOS and the PI3K/AKT signaling pathway (Deregibus et al., 2007).

Tumor cells can also hijack the normal vasculature and stimulate the rapid formation of new blood vessels to supply the tumor with nutrients (Carmeliet, 2005; Al-Nedawi et al., 2009; Grange et al., 2011). Pioneer studies in tumor angiogenesis focused on the whole cargo of EVs, including mRNA and proteins such as VEGF, FGF, IL-6, and IL-8 (Skog et al., 2008) that could stimulate the angiogenic phenotype in glioblastoma. Other proteins induced the same phenotype in an indirect manner in lung cancer (Al-Nedawi et al., 2009). The expression of the oncogenic epidermal growth factor receptor (EGFR) molecule on EVs is transferred to ECs, triggering the expression VEGF and contributing to an overall increased angiogenesis.

In general terms, angiogenesis is promoted by the transfer of mRNAs related to cell cycle progression and EC proliferation. For instance, EVs derived from the colorectal cancer cell line SW480 were enriched with M phase-related transcripts such as *CENPE*, *KIF15*, *CEP55*, *CCNA2*, *NEK2*, *PBK*, and *CDK8*, which initiated angiogenesis *via* stimulation of EC proliferation (Hong et al., 2009).

Overall, the hypoxic tumor microenvironment induces tumor cells to increase the release of EVs containing molecules that induce EC proliferation and facilitate angiogenesis. The new vasculature supplies nutrients and oxygen to cancer cells and provides an escape route for tumor cell migration and metastasis. An example of this process is a population of renal carcinoma cells that were found to secrete EVs containing the proangiogenic *VEGF, FGF2, Angiopoietin1, Ephrin A3, MMP2*, and *MMP9* mRNAs, which promoted *in vivo* angiogenesis and lung metastasis (Grange et al., 2011).

#### Immunosuppression

Extracellular vesicles mediate a wide range of immunological processes. Indeed, the first function of EVs described, contrary to the previous assumption that EVs were merely cell waste, was their function as antigen-presenting vesicles to recipient T cells (Raposo et al., 1996; Théry et al., 1999). Subsequent studies revealed that EVs participate in multiple activities, including T cell activation (Skokos et al., 2001; Taylor and Gerçel-Taylor, 2005), dendritic cell maturation (Skokos et al., 2003), and antigen presentation and macrophage activation (Schorey and Bhatnagar, 2008). The specific biological function depends on the immune cell type. For instance, EVs from B lymphocytes have potential antitumor effects, since they are potent immunostimulators. However, patients with cancer, especially those with advanced disease, often have depressed antitumor immunity (Penn, 1994). To grow, cancer cells are capable of modulating immune responses, which typically cause suppression of the immune system (Skokos et al., 2001; Taylor et al., 2003; Taylor and Gerçel-Taylor, 2005; Wen et al., 2016). Several mechanisms, including inactivation of the cytotoxic activity of T lymphocytes or natural killer (NK) cells, contribute to the ability of tumor cells to escape the immune response (Huang-Ge and William, 2012). NK cells are cytotoxic lymphocytes of the innate immune system that kill cancer cells directly (Liu et al., 2006; Vivier et al., 2017). However, under contact with EVs derived from murine mammary tumors, their cytolytic activity was reduced by a decrease in *perforin* gene expression, and IL-2 stimulated cell growth, allowing the implanted tumors to grow rapidly (Liu et al., 2006). Induction of T cell apoptosis by cancer cell-derived EVs has also been found in cancer (Taylor and Gerçel-Taylor, 2005; Muller et al., 2016). Ovarian cancer EVs carry the death ligands FasL and tumor necrosis factor–related apoptosis-inducing ligand (TRAIL), which cause significant DNA fragmentation and trigger the apoptotic pathway in T cells (Taylor et al., 2003; Jeong et al., 2005; Taylor and Gerçel-Taylor, 2005). Other mechanisms of immunosuppression include inhibition of cell differentiation, such as the differentiation of monocytes into dendritic cells (Valenti et al., 2007). This differentiation, in turn, is necessary for T cell activation. Since cancer cell-derived EVs block cell maturation, those cells instead become immunosuppressive cells by secreting factors that inhibit T lymphocyte proliferation, thus facilitating tumor progression (Wieckowski and Whiteside, 2006; Liu et al., 2010).

The crosstalk between cancer cells and immune cells *via* EVs has been gaining importance and is currently widely studied. However, most studies have focused on EV membrane proteins (Jeong et al., 2005; Taylor and Gerçel-Taylor, 2005; Muller et al., 2016), partially neglecting the role of packaged mRNAs in inhibiting the immune system. While it has been reported that T cells specifically show very low uptake of EVs, suggesting that the main effect of EVs is mediated by receptor binding on the surface membrane, other cells such as B lymphocytes and monocytes highly internalize EVs (Muller et al., 2016). Accordingly, the role of EV mRNA in monocytes has been described.

##### EVs in monocytes

Tumor-derived EVs contain mRNAs encoding growth factors, including *VEGF, HGF, IL-8*, and *CD44H*. When EVs are phagocytosed by monocytes, these molecules are transferred and activate the Akt signaling pathway, resulting in altered biological activity (Baj-Krzyworzeka et al., 2006).

##### EVs in T cells

Although most reports have focused on membrane proteins of EVs, a report suggest the biological effects of mRNAs contained in EVs on T cells. Under non-pathological conditions, T cell responses are critical for antitumor immunity. However, EVs diminish the proliferation and activation and increase the apoptosis of T cells, ultimately resulting in progressive loss of T cell function, a process called T cell exhaustion (Yi et al., 2010; Liu et al., 2015). Muller et al. (2016) reported alterations in the recipient T cell transcriptome upon treatment with cancer cell-derived EVs. An increase in the levels of several immunosuppressive genes and the levels of the corresponding proteins was found. Moreover, decreased levels of CD69, a T cell activation marker, were observed, suggesting that mRNA transcripts delivered by EVs to recipient T cells caused immunosuppression (Muller et al., 2016).

### Tumor Progression by Cancer Cell-to-Cancer Cell Transmission

Cancer cell-derived EVs are taken up by noncancerous cells and facilitate tumor progression by remodeling the microenvironment, promoting a more favorable environment for growth, as explained previously. However, it has also been shown that malignant cancer cells secrete EVs that can be taken up by less malignant cancer cells and affect their behavior. These behaviors often involve the acquisition of a more migratory and invasive phenotype.

In breast cancer, it was found that when the less-malignant T47D cells were located close to the highly malignant MDA-MB-231 cells, T47D cells migrated faster. Moreover, *via* the Cre-Lox system, it was shown that this increased motility was restricted to cells that had taken up MDA-MB-23-derived EVs, as shown by the switch in fluorescence (from red to green) (Zomer et al., 2015). Further analyses showed that this behavior was consistent with the EV cargo, which was enriched with migration-inducing mRNAs (Zomer et al., 2015), although the exact mRNAs were not specified in the original article. Because activation of migration and invasion often results in acquisition of a more metastatic phenotype (Nguyen et al., 2009), it is not surprising that the transfer of migration-inducing mRNAs also increases the metastatic potential of tumor cells. As a result, T47D cells that had taken up EVs released by the more malignant cell line and showed a more migratory phenotype also showed an increase in metastatic potential. These cells were 52-fold more abundant in the lungs than control cells (Zomer et al., 2015). Other studies also showed the increased invasive capacity of cancer cells upon contact with EVs derived from more metastatic cells. Malignant ovarian cancer cells expressing the stemness marker LIN28A promoted the invasion of HEK293 cells by activating epithelial-to-mesenchymal transition (Enriquez et al., 2015). Interestingly, it has been described that EVs derived from not only highly malignant cancer cells but also drug-resistant cells are able to induce migratory and invasive behaviors. Transfer of EVs derived from icotinib-resistant lung cancer cells into icotinib-sensitive lung cancer cells induced the expression of *MET*, which plays a role in migration. *MET* expression was correlated with increased migration and invasion of lung cancer cells (Yu et al., 2019).

The potential of cancer cells for invasion and migration is also dependent on their ability to degrade the ECM in which they are anchored. Accordingly, highly metastatic hepatocellular carcinoma cells induced migratory and invasive behaviors of nonmotile hepatocytes, providing the ability to degrade the ECM. EVs carrying *CAV1, CAV2*, and *S100A4* mRNA molecules, upon internalization, induced the PI3K/AKT and MAPK signaling pathways and eventually increased the secretion of the matrix-degrading proteases MMP-2 and MMP-9 (He et al., 2015). Other studies have shown that some cancer cell-derived EVs carry both the protein and mRNA of metalloproteinases such as *MMP1, MMP2*, and *MMP9* (Dolo et al., 1999; Hakulinen et al., 2008; He et al., 2015; Yokoi et al., 2017).

### Premetastatic Niche Formation

Metastasis is the ultimate consequence of cancer propagation and remains the leading cause of cancer-related mortality (Weigelt et al., 2005). However, to metastasize, tumor cells must manipulate the microenvironment in distant organs to optimize the conditions for the establishment and further growth of metastatic cells in a so-called premetastatic niche (Hood et al., 2011; Peinado, 2012; Sleeman, 2012; Chin and Wang, 2016; Costa-silva et al., 2018). These processes support cancer cell engraftment and survival (Sceneay et al., 2013). Since EVs are nanoparticles secreted into the extracellular space, they often enter body fluids circulating throughout the body. This precise systemic distribution of EVs and their capacity to alter the behavior of multiple types of cells have been shown to promote cancer metastasis, mainly *via* their contribution to generating a premetastatic niche (Hood et al., 2011; Peinado, 2012). Cancer cell-derived EVs contribute directly to this event by remodeling and degrading the ECM and organ parenchyma (Mu et al., 2013; Yokoi et al., 2017; Costa-silva et al., 2018) and *via* the recruitment of cells that promote metastasis, such as bone marrow-derived cells and macrophages (Peinado, 2012; Costa-silva et al., 2018), creating a microenvironment conducive to metastasis.

A clear example was described by Yokoi et al. (2017), who described the mechanisms of peritoneal metastasis promotion by EVs. Highly metastatic ovarian cancer cells secrete EVs with high concentrations of *MMP1* mRNA and are transferred to mesothelial cells, the most prevalent cells in the peritoneal cavity. Upon uptake, *MMP1* induces apoptosis in mesothelial cells, breaking the mesothelial-peritoneal barrier and promoting metastasis *in vivo*. Cells with knockdown of nSMase2, a protein required for EV secretion (Kosaka et al., 2013a), had a significant reduction in metastasis in the peritoneal cavity, although no major difference was found in the primary tumor size (Yokoi et al., 2017).

Moreover, Hood et al. (2011) demonstrated that premetastatic niche formation by EVs is a selective phenomenon mediated by the generation of liposomes to mimic empty EVs (these liposomes are nanovesicles lacking proteins, mRNAs, and miRNAs) but with a lipid content similar to that of the membrane. Their experiments showed the selective homing of melanoma EVs to sentinel lymph nodes and nonselective homing by liposomes (Hood et al., 2011). Moreover, EVs recruited melanoma cells to sentinel lymph nodes by altering the ECM, which promoted the trapping of melanoma cells within sentinel nodes. These studies helped to explain metastatic organotropism of melanoma. However, in most cancer types, the specificity of the metastatic organ cannot be explained by the anatomy of the blood and lymphatic circulatory networks (Fidler, 2003). Organotropism of EVs derived from cancer cells is logical if we take into consideration the internalization of EVs, which depends mainly on their structure and composition. Therefore, it is reasonable to think that organotropism is regulated mainly by molecules in the EV membrane. For instance, some integrins were found to be responsible for organotropism. EVs expressing integrin αvβ5 were associated with liver metastasis, while EVs expressing α6β4 and α6β1 were associated with lung metastasis (Hoshino et al., 2015). Since mRNAs have been found mainly inside EVs, we expect that these mRNAs have almost no function in organotropism. However, once EVs are taken up, their contained mRNAs play key roles in metastasis.

## Therapeutics

The discovery of EVs and their function allowed us to address one more layer of complexity in cancer. Thus, subsequent research should be directed at the use of this knowledge against cancer cells. One strategy is the use of EVs as biomarkers for cancer diagnosis. Another strategy is to use them as a therapeutic treatment. Both options will be further discussed below.

### Biomarkers

Currently, there is a need for biomarkers that can be utilized to diagnose cancers accurately and to predict drug sensitivities effectively (Collins and Varmus, 2015). A promising new approach for cancer diagnosis is focused on the use of biomarkers available in body fluids and able to be assessed in a noninvasive manner. In this area, EVs have great potential as biomarkers for cancer therapeutic monitoring since they are easily accessible, and their contents have been shown to be enriched with key molecules characteristic of the cells of origin (Atay and Godwin, 2014; Melo et al., 2015).

While it is true that most cells in the body secrete EVs, it has been demonstrated that tumors are especially potent producers of EVs; thus, cancer cell-derived EVs can be detected. Moreover, EVs have been found not only in blood (Caby et al., 2005; Dong et al., 2019) but also in most, if not all, body fluids, including urine (Pisitkun et al., 2004; Murakami et al., 2018), saliva (Yang et al., 2014; Han et al., 2018), cerebrospinal fluid (Chen et al., 2013; Akers et al., 2016), seminal fluid (Aalberts et al., 2012; Höög and Lötvall, 2015), breast milk (Admyre et al., 2007), amniotic fluid (Asea et al., 2008), ascites (Andre et al., 2002; Yokoi et al., 2017), and bile (Masyuk et al., 2010). Thus, EVs from most body fluids are a source for noninvasive or minimally invasive diagnosis of different cancers and, subsequently, as a tool for longitudinal disease monitoring (Collins and Varmus, 2015; Di Meo et al., 2017).

As mentioned previously, mRNA encapsulated within the phospholipid bilayer structure of EVs is protected from the harsh external environment, which would otherwise degrade it (El-Hefnawy et al., 2004; Skog et al., 2008; Cheng et al., 2014; Kim et al., 2017). Furthermore, in comparison with other EV cargo molecules, mRNA is very suitable as a biomarker due to the high sensitivity of PCR detection. Although it has been shown that the mRNA cargo in EVs is not an exact copy of the mRNA content in the cell of origin and that some mRNAs are enriched while others are depleted, the transcriptomic profile of EVs still reflects that of the cell of origin (Jenjaroenpun et al., 2013). Indeed, the transcriptomic signatures of EVs can differentiate healthy controls and patients with certain types of cancer, demonstrating their potential value as biomarkers (Noerholm et al., 2012).

#### Biomarkers for Cancer Diagnosis

Glioblastoma is a type of cancer that is very difficult to biopsy and requires invasive and dangerous brain surgery; thus, a biomarker for this kind of tumor is greatly required. The most prominent study in GBM found a variant of the gene EGFR, *EGFRvIII*, to be enriched in EVs isolated from patients, as has been described in 30% of GBM tumors (Johnson et al., 2012). *EGFRvIII* mRNA was easily detected by RT-PCR in patient serum (Skog et al., 2008). Other studies reported *MDK*, *COX8C*, *GALR3*, and *LOC653602* to be enriched in EVs from GBM patients (Skog et al., 2008).

Biomarkers in urine from patients with bladder cancer are another promising approach. Due to the high recurrence rate of bladder cancer, which is higher than 50% (Botterman et al., 2003), a noninvasive lifelong monitoring biomarker is needed. A study conducted on EVs secreted during several stages of urothelial cancer narrowed the field of promising urinary EV biomarkers to three mRNA molecules, *SLC2A1*, *GPRC5A*, and *KRT17*. Importantly, the performance of these biomarkers was equivalent to that of conventional urine cytology and bladder tumor antigen assays (Murakami et al., 2018).

Of special interest in the finding of salivary EVs derived from cancer cells is their potential for identification by a routine sampling method that would be even less invasive than blood analysis. Human saliva is a slightly acidic biofluid (pH 6.0–7.0) that contains many proteins, enzymes, bacteria, and unique mRNAs, hindering the identification of biomarkers (Li et al., 2004; Lee and Wong, 2009). However, several studies have succeeded in discovering saliva-based biomarkers for oral and systemic cancers (Li et al., 2004). Yang et al. (2014) demonstrated the presence of distal cancer EVs in mouse saliva. To this end, they generated a lung cancer model in which cancer cells constitutively expressed the hCD63-GFP protein. The authors identified human *CD63-GFP*-containing EVs and *GAPDH* mRNA molecules in the blood and saliva of tumor-bearing mice (Yang et al., 2014). Furthermore, the lung cancer-specific mRNA molecules *BRAF*, *EGFR*, *LZTS1*, and *FGF19* were also found in the isolated salivary EVs (Yang et al., 2014). In addition, other researchers have reported the presence of human pancreatic cancer markers in salivary EVs (Lau et al., 2013), suggesting that the presence of cancer EVs in saliva may be a common factor in cancers that should be properly addressed in the future.

Ascites is a condition commonly found in patients with gastric and ovarian cancers and consists of fluid accumulation in the peritoneal cavity. This fluid has been described to contain cancer cells and promote metastasis. Moreover, EVs derived from cancer cells have been found (Yokoi et al., 2017). These EVs contained high levels of *MMP1* mRNA that induced apoptosis in mesothelial cells (Yokoi et al., 2017) *MMP1* expression levels in EVs from ascites fluid were strongly related to patient prognosis in early stages of ovarian cancer and thus are a promising biomarker for the degree of ovarian cancer malignancy.

Many studies have been performed on cell lines as preliminary screens. For instance, EVs extracted from conditioned medium of several hepatocellular carcinoma cell lines were found to have a rather large variation in the candidate mRNAs (Berardocco et al., 2017). Among the mRNAs, *TGM2* was contained in all EV cell lines and is a candidate for use as a hepatocellular carcinoma marker. Similarly, in prostate cancer cell lines, the expressed mRNAs in cancer cell-derived EVs were characteristic of those in the parental cells, especially with the differential expression of androgen metabolism genes such as *ETV1* and *FASN* (Shen and Abate-Shen, 2010; Huang et al., 2016), suggesting the use of those markers to distinguish the androgen sensitivity of prostate cancer (Lázaro-Ibáñez et al., 2017). Studies in cell lines, as previously shown, have provided insight into possible biomarkers, but these results still have to be confirmed in patients to ascertain the real potential of these molecules as biomarkers. As an example, Cha et al. (2020) selected a total of 12 mRNAs that were differentially expressed in EVs from 4 colorectal cancer cell lines, and through a series of comparisons, identified that the combination of only two of these mRNAs—*CD133* and *VEGF*—was able to distinguish colorectal cancer patients from healthy controls. However, the combination of these two mRNAs achieved 100% sensitivity with extremely high specificity and accuracy.

To date, numerous EV mRNAs have been reported as specific cancer biomarkers in diverse cancer types and body fluids, especially serum. An extensive list of these mRNAs is provided in Table 2.

**TABLE 2:** Summary of several cancer-associated biomarkers and the derived biofluid.

**mRNA**	**Type of cancer**	**Biofluid**	**Specificities**	**References**
*EGFRvIII*	Glioblastoma	Serum		Skog et al., 2008
*MDK, COX8L, GALR3, LOC653601*	Glioblastoma	N.S.		Skog et al., 2008
*SLCA1, GPRC5A, KRT7*	Bladder cancer	Urine		Murakami et al., 2018
*BRAF, EGFR, LZT5, FGF19*	Lung cancer	Saliva		Yang et al., 2014
*Apbb1ip, Aspn, Incenp, Daf2, Foxp1, BCO31781, Gng2*	Pancreatic cancer	Saliva	Mouse model.	Lau et al., 2013
*MMP1*	Ovarian cancer	Ascites	Capable to differentiate cancer-stages.	Yokoi et al., 2017
*TGM2*	Hepatocellular cancer	Cell culture medium		Berardocco et al., 2017
*ETV1, FASN*	Prostate cancer	Cell culture medium	Capable to discriminate androgen sensitivity.	Shen and Abate-Shen, 2010; Huang et al., 2016; Lázaro-Ibáñez et al., 2017
*CD133, VEGF*	Colorectal cancer	Cell culture medium		Cha et al., 2020
*PD-L1*	Melanoma, lung cancer	Plasma	Indicates the response to nivolumab and pembrolizumab treatments.	Del Re et al., 2018

*N.S., not specified.*

#### Biomarkers for Treatment Prognosis

Cancer EVs can be used not only as a diagnostic tool but also as a prognostic factor for cancer treatment outcomes. For example, measurement of *PD-L1* mRNA in plasma-derived EVs from melanoma and non-small cell lung cancer patients allowed monitoring of the therapeutic response to nivolumab and pembrolizumab, agents that are anti-PD-1 antibodies (Del Re et al., 2018). Patients who responded to the treatment presented EVs with decreased *PD-L1* mRNA levels compared to initial levels, while no difference was found in nonresponding patients. Monitoring of implanted treatments is very useful to identify the patient population responding to the treatment and to adjust the treatment of no-responding patients to achieve a proper treatment regimen.

### Therapeutic Tools

Multiple important functions by which cancer cell-derived EVs promote their growth and metastasis through both the local and systemic transfer of genetic material have been described. Thus, suppression of EVs impairs the metastatic ability of cancer cells. Moreover, similar to the utility of EVs as biomarkers, their ability to circulate systemically throughout the human body makes EVs an interesting target for novel therapeutic approaches.

#### Inhibition of EVs as a Therapeutic Approach

Because EVs play roles in multiple biological functions, including invasion, migration, angiogenesis, immunosuppression, and metastasis promotion, one potential strategy is to reduce the release of EVs from tumor cells or deplete them from the circulation. Although the complete mechanism of exosome release is not fully understood, it is known that the Rab GTPases Rab27a and Rab27b, as well as ceramides (e.g., nSMase2), play important roles in regulating exosome secretion (Ostrowski et al., 2010; Kosaka et al., 2013a). Knockdown of nSMase2 in cancer cells was found to reduce the level of EV secretion. Importantly, the metastatic ability of breast and ovarian cancer cells *in vivo* was dramatically reduced (Kosaka et al., 2013a; Yokoi et al., 2017).

On the other hand, depletion of circulating EVs has been attempted by dialysis (Marleau et al., 2012) and antibody targeting. Nishida-Aoki et al. (2017) took advantage of the opsonization effect of macrophages. When human-specific anti-CD9 or anti-CD63 antibodies were administered in a mouse model, macrophages phagocytized human cancer cell-derived EVs, and lung metastasis was significantly decreased. Although the results were promising, those experiments were performed in mouse models and used human antibodies. Thus, this methodology is not suitable for direct use in humans, since CD9 and CD63 are ubiquitous in the human body, and their eradication would generate several side effects (Nishida-Aoki et al., 2017). Future approaches should be directed toward cancer-specific molecules (Raposo and Stoorvogel, 2013), which were also found in EVs. This approach has been proposed as well by Zaborowski et al. (2019), who developed a methodology to identify such markers.

#### Use of Non-pathological mRNAs Carried by EVs

Because EVs are taken up by cancer cells, an alternative therapeutic approach is to reengineer naturally derived EVs for targeted gene therapy and drug delivery (Arslan et al., 2013; Katsuda et al., 2013; Ohno et al., 2013; Mao et al., 2018). Furthermore, exosomes have a long circulating half-life, and the lipid bilayer protects the mRNA cargo from degradation, facilitating its delivery to the target cells (Vickers and Remaley, 2012). For instance, *ECRG4* is a tumor suppressor gene commonly downregulated in many cancers, including breast, prostate, lung, liver, colon, brain, and head and neck cancers (Götze et al., 2009; Li et al., 2009; Xu et al., 2013). Transfer of *ECRG4* mRNA by EVs into human tongue squamous carcinoma cells increased its basal expression level and eventually inhibited malignant phenotypes *in vitro* and suppressed tumor growth and metastasis *in vivo* (Mao et al., 2018). To obtain EVs with high levels of *ECGR4*, the authors previously overexpressed this gene in HEK293 cells, from which they obtained the EVs. Overexpression in a cell line such as HEK293 may not be a recommended method for clinical use due to poor control of the complete EV cargo. Thus, the use of this method for clinical purposes is controversial. Moreover, there are other issues facing EV therapy. The main one is the process of gene loading into EVs, which has not been very successful to date. Electroporation, the main approach, usually results in fragmented mRNA (Hung and Leonard, 2016). To overcome this problem, novel approaches must be studied. A different method was offered by Bolukbasi et al. (2012), who found a 25-nt zip code-like sequence in mRNA that promoted encapsulation within EVs (discussed previously). Addition of this zip code in the 3′UTR of a specific mRNA resulted in enrichment of this mRNA in EVs (Bolukbasi et al., 2012). The use of this zip code or a similar one that promotes the loading of a specific mRNA into EVs for their later use in cancer treatment is very promising.

## Conclusion and Future Directions

Messenger RNA in EVs provides an extensive resource for biomarkers that are highly sensitive and easily accessible in body fluids. Due to encapsulation within the lipid bilayer membrane, mRNA molecules remain stable in the circulation and are a promising tool for cancer diagnosis. Moreover, both native and modified EVs can be used as biological therapeutics. However, although many biomarkers have been reported, especially in cell lines, their function is often neglected. mRNA carried by EVs can be translated into proteins within target cells, inducing several phenotypes that are beneficial for cancer cells. Considering the functions of mRNAs in EVs that are already understood, including the modulation of tumorigenesis and the tumor microenvironment by the promotion of angiogenesis, the immune response, drug resistance and invasion, as well as the remodeling at distant sites that induces metastasis, we expect that EV-derived mRNAs perform many additional important functions in cancer pathogenesis. We believe that an understanding of these molecules will provide a novel target for anticancer therapies.

## Author Contributions

MP-V: conceptualization and writing. YY and TO: review and supervision. All authors contributed to the article and approved the submitted version.

## Conflict of Interest

The authors declare that the research was conducted in the absence of any commercial or financial relationships that could be construed as a potential conflict of interest.

## Publisher’s Note

All claims expressed in this article are solely those of the authors and do not necessarily represent those of their affiliated organizations, or those of the publisher, the editors and the reviewers. Any product that may be evaluated in this article, or claim that may be made by its manufacturer, is not guaranteed or endorsed by the publisher.
